# Exclusion of emphysematous lung from dose-volume estimates of risk improves prediction of radiation pneumonitis

**DOI:** 10.1186/s13014-017-0891-z

**Published:** 2017-10-02

**Authors:** Yasuki Uchida, Takuya Tsugawa, Sachiko Tanaka-Mizuno, Kazuo Noma, Ken Aoki, Wataru Shigemori, Hiroaki Nakagawa, Daisuke Kinose, Masafumi Yamaguchi, Makoto Osawa, Emiko Ogawa, Yasutaka Nakano

**Affiliations:** 1 Division of Respiratory Medicine, Department of Internal Medicine, Shiga University of Medical Science, Otsu, Shiga Japan; 2 Department of Radiology, Shiga University of Medical Science, Otsu, Shiga Japan; 3 Department of Medical Statistics, Shiga University of Medical Science, Otsu, Shiga Japan; 4 The Center for Data Science Education and Research, Shiga University, Hikone, Shiga Japan; 5 Department of Radiology, Shiga University of Medical Science Hospital, Otsu, Shiga Japan; 6 Division of Infection Control and Prevention, Shiga University of Medical Science Hospital, Otsu, Shiga Japan; 7 Health Administration Center, Shiga University of Medical Science, Otsu, Shiga Japan

**Keywords:** Radiation pneumonitis, Chronic obstructive pulmonary disease, Low attenuation volume, Dosimetric parameter, Lung cancer

## Abstract

**Background:**

The risk factors for radiation pneumonitis (RP) in patients with chronic obstructive pulmonary disease (COPD) are unclear. Mean lung dose (MLD) and percentage of irradiated lung volume are common predictors of RP, but the most accurate dosimetric parameter has not been established. We hypothesized that the total lung volume irradiated without emphysema would influence the onset of RP.

**Methods:**

We retrospectively evaluated 100 patients who received radiotherapy for lung cancer. RP was graded according to the Common Terminology Criteria for Adverse Events (version 4.03). We quantified low attenuation volume (LAV) using quantitative computed tomography analysis. The association between RP and traditional dosimetric parameters including MLD, volume of the lung receiving a dose of ≥2 Gy, ≥ 5 Gy, ≥ 10 Gy, ≥ 20 Gy, and ≥30 Gy, and counterpart measurements of the lung without LAV, were analyzed by logistic regression. We compared each dosimetric parameter for RP using multiple predictive performance measures including area under the receiver operating characteristic curve (AUC) and integrated discrimination improvement (IDI).

**Results:**

Of 100 patients, RP of Grades 1, 2, 3, 4, and 5 was diagnosed in 24, 12, 13, 1, and 1 patients, respectively. Compared with traditional dosimetric parameters, counterpart measurements without LAV improved risk prediction of symptomatic RP. The ratio of the lung without LAV receiving ≥30 Gy to the total lung volume without LAV most accurately predicted symptomatic RP (AUC, 0.894; IDI, 0.064).

**Conclusion:**

Irradiated lung volume without LAV predicted RP more accurately than traditional dosimetric parameters.

**Electronic supplementary material:**

The online version of this article (10.1186/s13014-017-0891-z) contains supplementary material, which is available to authorized users.

## Background

Smoking is a major cause of both lung cancer and chronic obstructive pulmonary disease (COPD). Whether emphysematous lesion is a risk factor for radiation pneumonitis (RP) after radiotherapy (RT) is an important clinical problem, but the results obtained so far have been controversial. Some studies showed that COPD is a risk factor for RP [1–4], while others reported that RP was milder in patients with more severe COPD than in patients with normal lung function [5, 6]. There was also a report that COPD does not influence RP [7]. In that study, a scoring system similar to the modified Goddard system was used to evaluate the association between RP and emphysema [7], but could not assess the potential influence of emphysematous lesions within the irradiation field on RP. The relationship between RP and emphysematous lesions within the irradiation field has not been examined.

Mean lung dose (MLD) and the percentage of lung volume receiving 20 Gy or more (V20%) are the most commonly recognized traditional dosimetric parameters associated with risk for RP [8–13]. However, a best dosimetric parameter for RP has not been determined.

The purposes of this study were twofold. First, to elucidate whether COPD is a risk factor for RP after RT in lung cancer patients, we examined the relationship between RP and dosimetric parameters associated with emphysematous lesions within the irradiation field. Second, we compared their predictive performances to determine which dosimetric parameter had the most predictive ability for RP.

## Materials and Methods

### Selection of participants

Patients who received RT for lung cancer at our institution between June 2010 and July 2015 (*N* = 100) were retrospectively selected. Inclusion criteria were predefined as follows: first time receiving RT; total irradiation dose >30 Gy; pneumonectomy not performed within 5 months after the RT or before the occurrence of symptomatic RP; follow-up period >5 months if symptomatic RP did not occur; and entire lung fields scanned using computed tomography (CT) before RT.

### Radiotherapy planning and image analysis

**Fig. 1:**
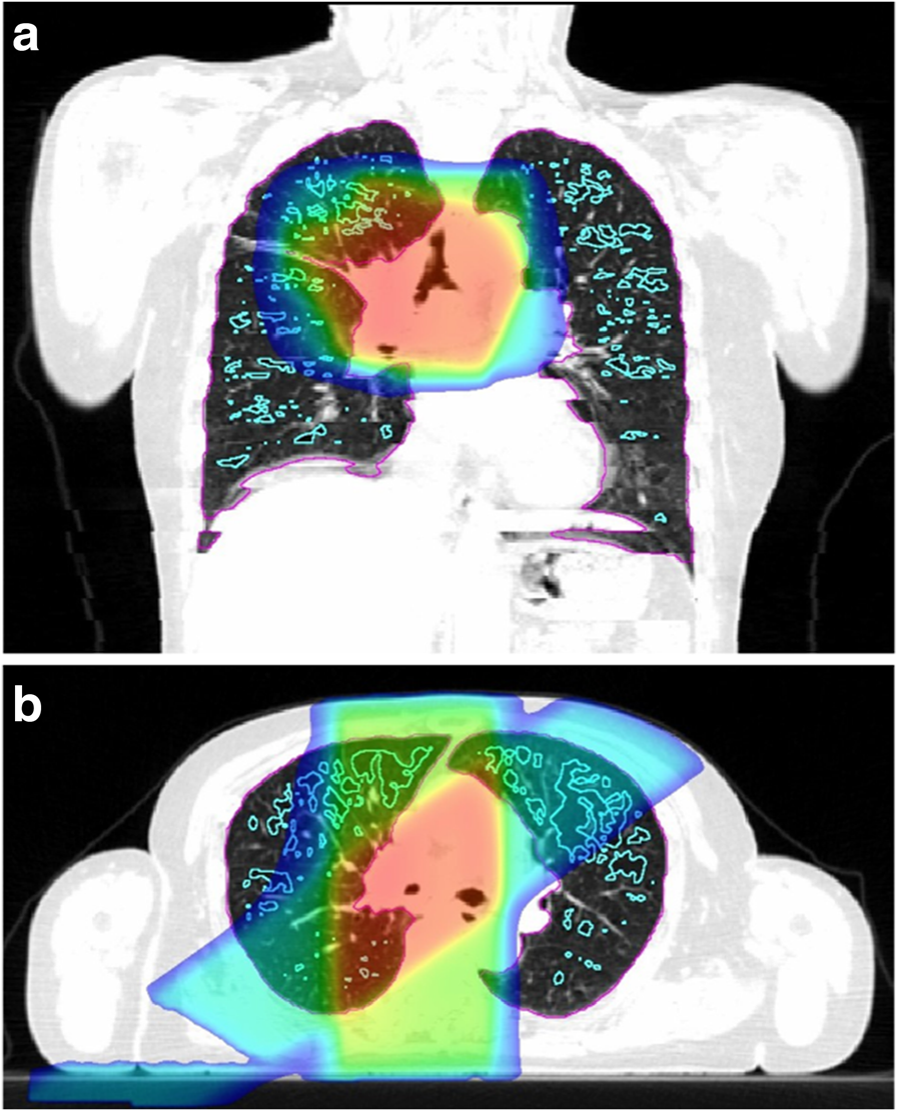
The area inside the light blue line was the LAV and the threshold was −856 HU. The area inside the purple line is the lung. The colorful area (for example red, yellow, green and blue) was the irradiated area and the overlaps were calculated

RT planning was done using Eclipse™ software (Varian Medical Systems, Palo Alto, CA) with an analytical anisotropic algorithm. The distribution of radiation dose was calculated using lung heterogeneity corrections. Patients were treated with curative or palliative intent with RT alone or with concurrent chemoradiation. Ninety-five patients (Ninety-five percent) were treated with 3D conformal RT and five patients (5 %) were treated with intensity-modulated RT. The total dose varied between 30 Gy and 66 Gy. CT scans were undertaken under free breathing before RT. Low attenuation volume (LAV), which represents emphysematous lesions in the lung, was evaluated using the threshold limit of −856 HU (Fig. 1a,b) [14]. We validated the association between the CT under free breathing and the inspiratory CT performed within 45 days after free breathing CT. Inspiratory CT was performed using Toshiba Aquillion ONE (Toshiba Medical Systems Corp., Otawara, Tochigi, Japan) and LAV was analyzed using Aquarius iNtuition™ software ver.4.4.12 (TeraRecon Inc., San Mateo, Calif) and evaluated using the threshold limit of −950 HU. LAV was evaluated in both the right and left lungs. We also measured total lung volume (TLV) from the CT data, and the ratio of LAV to TLV (LAV%) was calculated. The mean emphysema dose (MED) was defined as the mean dose of the irradiated LAV, and the mean lung without emphysema dose (MLWED) was defined as the mean dose of the irradiated TLV without LAV. The dosimetric parameters we evaluated were listed in Table 1.

**Table 1:** Evaluated dosimetric parameters

Parameter	
V2 (cc)	The volume of the lung receiving a dose ≥2 Gy
V5 (cc)	The volume of the lung receiving a dose ≥5 Gy
V10 (cc)	The volume of the lung receiving a dose ≥10 Gy
V20 (cc)	The volume of the lung receiving a dose ≥20 Gy
V30 (cc)	The volume of the lung receiving a dose ≥30 Gy
V2 − LAV2 (cc)	The volume of the lung without LAV receiving a dose of ≥2 Gy
V5 − LAV5 (cc)	The volume of the lung without LAV receiving a dose of ≥5 Gy
V10 − LAV10 (cc)	The volume of the lung without LAV receiving a dose of ≥10 Gy
V20 − LAV20 (cc)	The volume of the lung without LAV receiving a dose of ≥20 Gy
V30 − LAV30 (cc)	The volume of the lung without LAV receiving a dose of ≥30 Gy
LAV2 (cc)	The volume of LAV receiving a dose of ≥2 Gy
LAV5 (cc)	The volume of LAV receiving a dose of ≥5 Gy
LAV10 (cc)	The volume of LAV receiving a dose of ≥10 Gy
LAV20 (cc)	The volume of LAV receiving a dose of ≥20 Gy
LAV30 (cc)	The volume of LAV receiving a dose of ≥30 Gy
V2%	The percentage of lung volume receiving ≥2 Gy
V5%	The percentage of lung volume receiving ≥5 Gy
V10%	The percentage of lung volume receiving ≥10 Gy
V20%	The percentage of lung volume receiving ≥20 Gy
V30%	The percentage of lung volume receiving ≥30 Gy
(V2 − LAV2)∕TLV	The ratio of the lung without LAV receiving ≥2 Gy to the TLV
(V5 − LAV5)∕TLV	The ratio of the lung without LAV receiving ≥5 Gy to the TLV
(V10 – LAV10)∕TLV	The ratio of the lung without LAV receiving ≥10 Gy to the TLV
(V20 – LAV20)∕TLV	The ratio of the lung without LAV receiving ≥20 Gy to the TLV
(V30 – LAV30)∕TLV	The ratio of the lung without LAV receiving ≥30 Gy to the TLV
(V2 − LAV2)∕(TLV – LAV)	The ratio of the lung without LAV receiving ≥2 Gy to the TLV without LAV
(V5 – LAV5)∕(TLV – LAV)	The ratio of the lung without LAV receiving ≥5 Gy to the TLV without LAV
(V10 – LAV10)∕(TLV – LAV)	The ratio of the lung without LAV receiving ≥10 Gy to the TLV without LAV
(V20 − LAV20)∕(TLV – LAV)	The ratio of the lung without LAV receiving ≥20 Gy to the TLV without LAV
(V30 – LAV30)∕(TLV – LAV)	The ratio of the lung without LAV receiving ≥30 Gy to the TLV without LAV
LAV (cc)	Low attenuation volume
LAV%	The ratio of LAV to the total lung volume
TLV – LAV (cc)	Total lung volume without LAV
MLD (Gy)	Mean lung dose
MED (Gy)	Mean dose of the irradiated LAV,
MLWED (Gy)	Mean lung without emphysema dose

### Clinical toxicity

Cases of RP were retrospectively monitored using the Common Terminology Criteria for Adverse Events, version 4.03 [15]. The primary endpoint for this analysis was symptomatic RP ≥ Grade 2, and the secondary endpoint was RP ≥ Grade 3. Patients were generally followed for 3 to 6 weeks after completion of RT, and at 3- to 6-month intervals thereafter. A diagnosis of RP was made on the basis of radiographic images, laboratory tests, physical examination, clinical symptoms, and medical records.

The study protocol was approved by the Institutional Review Board, which waived written informed consent because of the retrospective design.

### Statistical analysis

We used summary statistics to analyze clinical factors including age, sex, disease stage, histology type, type of RT, chemotherapy, smoking history, smoking index, body mass index (BMI), and interstitial lung disease (ILD) for all patients, and classified patients as symptomatic RP, and RP ≥ Grade 3. For description, we used median and range for continuous variables, and percentage for categorical variables. We also compared the clinical factors between RP ≥ Grade 2 and RP ≤ Grade 1, and between RP ≥ Grade 3 and RP ≤ Grade 2, using Wilcoxon’s rank sum test or Fisher’s exact test.

Traditional dosimetric parameters including MLD, V2%, V5%, V10%, V20%, and V30%, and other dosimetric parameters associated with LAV, were described with median and interquartile range.

Multivariable logistic regression was conducted to evaluate the association between each dosimetric parameter and the onset of symptomatic RP, or RP ≥ Grade 3. Dosimetric parameters were divided by the standard deviation of each. The adjusted factors were decided using the result of univariate analysis. The predictive performance of each dosimetric parameter for RP was compared using the area under the receiver operating characteristic curve (AUC), Akaike’s information criterion (AIC), Bayesian information criterion (BIC), integrated discrimination improvement (IDI), and the net reclassification improvement (NRI). For IDI and NRI, the model with MLD was used as a reference model. We required a *p* value <0.05 for statistical significance. Statistical analyses were performed using JMP version 11 (SAS Institute Inc., Cary, NC) and SAS software version 9.4 (SAS Institute Inc., Cary, NC).

## Results

### Clinical parameters

RP was observed in 51 out of 100 patients: Grade 1, 24 patients; Grade 2, 12 patients; Grade 3, 13 patients; Grade 4, one patient; and Grade 5, one patient. Forty-nine patients did not develop RP. Therefore, in total, 27 patients developed RP ≥ Grade 2 and 15 patients developed RP ≥ Grade 3.

The follow-up period after the onset of RP was between 1 and 60 months (median, 12 months). Six patients died from lung cancer or RP, and one patient was referred to another hospital, after the onset of symptomatic RP and within 5 months of receiving RT. The period of observed onset of symptomatic RP was between 2 days and 8 months (median, 1 month) after RT.

Tables 2 and 3 show the results of univariate analysis. In the univariate analysis, disease stage (Stage 3), chemotherapy, ILD, MLD, and V20% were significantly associated with the occurrence of symptomatic RP (Table 2). None of the chemotherapy regimens were significantly associated with the occurrence of symptomatic RP (*P* = 0.599). Thirty-six patients received chemotherapy and RT concurrently, and four patients received chemotherapy before RT. If we limit RP ≥ Grade 3, staging (Stage 3), chemotherapy, ILD, and histology type were significantly associated with the occurrence of RP in the univariate analysis (Table 3).

**Table 2:** Clinical parameters in symptomatic radiation pneumonitis patients and asymptomatic patients

Characteristic	Total No. of Patients (*N* = 100)	No. of Symptomatic Patients (≥ grade 2 RP) (*N* = 27)	No. of Asymptomatic Patients (≤ grade 1 RP) (*N* = 73)	*P* Value
Median age (range), y	72 (39–89)	70 (59–82)	73 (39–89)	0.248
Male sex	82 (82)	25 (92.6)	57 (80.3)	0.221
Disease stage				0.003
1	25 (25)	1 (3.7)	24 (32.9)	
2	9 (9)	2 (7.4)	7 (9.6)	
3	51 (51)	21 (77.8)	30 (41.1)	
4	15 (15)	3 (11.1)	12 (16.4)	
^a^ Histology type				0.060
SqCC	33 (33)	12 (44.4)	21 (28.8)	
Adenocarcinoma	27 (27)	5 (18.5)	22 (30.1)	
SCC	15 (15)	7 (25.9)	8 (11.0)	
NSCC	8 (8)	2 (7.4)	6 (8.2)	
Unknown	14 (14)	1 (3.7)	13 (17.8)	
Others	3 (3)	0 (0)	3 (4.1)	
Treatment type				0.103
IMRT	5 (5)	0 (0)	5 (6.8)	
3D Conformal	95 (95)	27 (100)	68 (93.2)	
Chemotherapy				<0.0001
Yes	40 (40)	20 (74.1)	20 (27.4)	
No	60 (60)	7 (25.9)	53 (72.6)	
Smoking history				0.196
Current	24 (24)	10 (37.0)	14 (19.2)	
Former	59 (59)	13 (48.2)	46 (63.0)	
Never	17 (17)	4 (14.8)	12 (17.8)	
Smoking (range), pack-years	40 (0–180)	45 (0–120)	36 (0–180)	0.195
Median BMI (range), kg/m^2^	20.3 (14.98–27.40)	20.55 (16.19–24.83)	20.16 (14.98–27.40)	0.395
ILD				0.0358
Yes	6 (6)	4 (14.8)	2 (2.7)	
No	94 (94)	23 (85.2)	71 (97.3)	
Surgery				0.3142
None	95 (95)	25 (92.6)	70 (95.9)	
Pre-RT	1 (1)	1 (3.7)	0 (0)	
Post-RT	4 (4)	1 (3.7)	3 (4.1)	
Median MLD (IQR), Gy	7.2 (3.676–10.572)	11.416 (8.615–16.801)	4.854 (3.338–8.146)	<0.0001
Median V20% (IQR)	13.554 (5.872–20.449)	21.153 (17.092–30.368)	10.314 (4.802–14.529)	<0.0001
Median LAV% (IQR)	0.103 (0.026–0.257)	0.095 (0.037–0.254)	0.108 (0.018–0.279)	0.907

(Wilcoxon’s rank sum test or Fisher’s exact test)

^a^Percentages in this column may not add up to exactly 100% because of rounding

Unless otherwise specified, data are expressed as numbers of patients, and numbers in parentheses are percentages. RP = radiation pneumonitis; SqCC = squamous cell carcinoma; SCC = small cell carcinoma; NSCC = non-small cell carcinoma; IMRT = intensity-modulated radiotherapy; BMI = body mass index; ILD = interstitial lung disease; MLD = mean lung dose; IQR = interquartile range; V20% = percentage of lung volume irradiated ≥20 Gy; LAV% = ratio of low attenuation volume to the lung volume

**Table 3:** Clinical parameters in patients with radiation pneumonitis ≥ Grade 3 and ≤ Grade 2

Characteristic	Total No. of Patients (*N* = 100)	No. of ≥ Grade 3 RP Patients (*N* = 15)	No. of ≤ Grade 2 RP Patients (*N* = 85)	*P* Value
Median age (range), y	72 (39–89)	71 (62–80)	72 (39–89)	0.988
Male sex	82 (82)	14 (93.3)	68 (81.9)	0.453
Disease stage				0.0398
1	25 (25)	0 (0)	25 (29.4)	
2	9 (9)	2 (13.3)	7 (8.2)	
3	51 (51)	11 (73.3)	40 (47.1)	
4	15 (15)	2 (13.3)	13 (15.3)	
^a^ Histology type				0.0479
SqCC	33 (33)	10 (66.7)	23 (27.1)	
Adenocarcinoma	27 (27)	2 (13.3)	25 (29.4)	
SCC	15 (15)	3 (20.0)	12 (14.1)	
NSCC	8 (8)	0 (0)	8 (9.4)	
Unknown	14 (14)	0 (0)	14 (16.4)	
Others	3 (3)	0 (0)	3 (3.5)	
Treatment type				1.000
IMRT	5 (5)	0 (0)	5 (5.9)	
3D Conformal	95 (95)	15 (100)	80 (94.1)	
^a^Chemotherapy				0.0082
Yes	40 (40)	11 (73.3)	29 (34.1)	
No	60 (60)	4 (26.7)	56 (65.9)	
Smoking history				0.174
Current	24 (24)	1 (6.7)	23 (27.1)	
Former	59 (59)	12 (80.0)	47 (55.3)	
Never	17 (17)	2 (13.3)	15 (17.6)	
Smoking (range), pack-years	40 (0–180)	52 (0–120)	39 (0–180)	0.118
Median BMI (range), kg/m^2^	20.30	20.91	19.98	0.126
	(14.98–27.40)	(16.19–24.83)	(14.98–27.40)	
^a^ILD				0.0420
Yes	6 (6)	3 (20.0)	3 (3.5)	
No	94 (94)	12 (80.0)	82 (96.5)	
Surgery				0.0783
No surgery	95 (95)	13 (86.7)	82 (96.5)	
Pre-RT	1 (1)	1 (6.7)	0 (0)	
Post-RT	4 (4)	1 (6.7)	3 (3.5)	
Median MLD (IQR), Gy	7.200	10.717	5.896	<0.0001
	(3.676–10.572)	(8.590–17.610)	(3.474–9.308)	
Median V20% (IQR)	13.554	20.522	12.626	<0.0001
	(5.872–20.449)	(15.336–27.924)	(5.039–18.328)	
Median LAV% (IQR)	0.103	0.085	0.112	0.798
	(0.026–0.257)	(0.035–0.254)	(0.021–0.260)	

(Wilcoxon’s rank sum test or Fisher’s exact test)

^a^Percentages in this column may not add up to exactly 100% because of rounding. Unless otherwise specified, data are expressed as numbers of patients, and numbers in parentheses are percentages. RP = radiation pneumonitis; SqCC = squamous cell carcinoma; SCC = small cell carcinoma; NSCC = non-small cell carcinoma; IMRT = intensity-modulated RT; BMI = body mass index; ILD = interstitial lung disease; MLD = mean lung dose; V20% = percentage of lung volume irradiated with ≥20Gy; IQR = interquartile range; LAV% = ratio of LAV to total lung volume

### Dose volume parameter

**Fig. 2:**
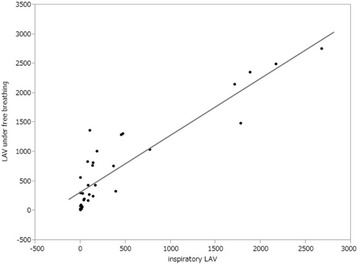
Spearman rank correlations between the LAV in inspiratory CT and the LAV in CT under free breathing in 33 patients. There was a significant relationship between these two measurements (LAV in CT under free breathing = 0.97 × LAV in inspiratory CT + 322.5, *r* = 0.839, *P* < .0001)

Thirty-three patients underwent inspiratory CT within 45 days after free-breathing CT under the same condition. The LAV in inspiratory CT was highly correlated with the LAV in CT under free breathing (Fig. 2). Since patients who received chemotherapy were almost equal to the patients of disease stage 3, chemotherapy and interstitial lung disease (ILD) were used as adjusted factors in the logistic models. Multivariable logistic regression analysis for symptomatic RP (≥Grade 2) demonstrated that none of the dosimetric parameters that included LAV (i.e. LAV2, LAV5, LAV10, LAV20, LAV30, LAV, LAV%, and TLV – LAV) were significantly related to symptomatic RP (Table 4). Irradiated lung volume (V2, V5, V10, V20, V30) and counterpart measurements of the lung without LAV (V2 – LAV2, V5 – LAV5, V10 – LAV10, V20 – LAV20, V30 – LAV30) all were significantly associated with the occurrence of symptomatic RP. Every irradiated lung volume measurement without an LAV parameter (V2 – LAV2, V5 – LAV5, V10 – LAV10, V20 – LAV20, V30 – LAV30) had lower *p* values and higher odds ratios than the counterpart values with LAV (V2, V5, V10, V20, V30), indicating a stronger association with symptomatic RP. The percentage of irradiated lung volume (V2%, V5%, V10%, V20%, V30%) and counterpart measurements of the lung without LAV were also significantly associated with the occurrence of symptomatic RP. The MLD, MED, and MLWED all were significantly associated with the occurrence of symptomatic RP; however, a comparison of *p* values and odds ratios between the three parameters suggested a stronger association between MLWED and the occurrence of symptomatic RP.

**Table 4:** Multivariate logistic regression analysis for symptomatic radiation pneumonitis (≥ Grade 2)

Parameter	Odds ratio	*P* Value
V2 (cc)	1.768 (1.056–3.104)	0.0358
V5 (cc)	1.795 (1.071–3.160)	0.0315
V10 (cc)	2.034 (1.196–3.710)	0.0122
V20 (cc)	2.270 (1.309–4.287)	0.0057
V30 (cc)	2.621 (1.482–5.055)	0.0018
V2 − LAV2 (cc)	2.037 (1.175–3.827)	0.0168
V5 − LAV5 (cc)	2.098 (1.192–4.054)	0.0164
V10 − LAV10 (cc)	2.451 (1.344–5.011)	0.0071
V20 − LAV20 (cc)	2.900 (1.525–6.228)	0.0028
V30 − LAV30 (cc)	3.627 (1.852–7.960)	0.0005
LAV2 (cc)	1.058 (0.618–1.718)	0.823
LAV5 (cc)	1.059 (0.615–1.702)	0.822
LAV10 (cc)	1.118 (0.650–1.775)	0.650
LAV20 (cc)	1.143 (0.666–1.791)	0.578
LAV30 (cc)	1.116 (0.640–1.742)	0.652
V2%	2.535 (1.422–4.935)	0.0030
V5%	2.421 (1.360–4.690)	0.0047
V10%	2.957 (1.586–6.188)	0.0016
V20%	3.771 (1.897–8.671)	0.0005
V30%	4.996 (2.392–12.299)	<0.0001
(V2 − LAV2)∕TLV	2.467 (1.384–4.792)	0.0040
(V5 − LAV5)∕TLV	2.376 (1.337–4.168)	0.0054
(V10 – LAV10)∕TLV	2.913 (1.558–6.096)	0.0019
(V20 – LAV20)∕TLV	4.085 (1.984–9.865)	0.0005
(V30 – LAV30)∕TLV	6.114 (2.726–16.873)	<0.0001
(V2 − LAV2)∕(TLV – LAV)	2.810 (1.551–5.621)	0.0015
(V5 – LAV5)∕(TLV – LAV)	2.788 (1.532–5.606)	0.0017
(V10 – LAV10)∕(TLV – LAV)	3.363 (1.756–7.632)	0.0008
(V20 − LAV20)∕(TLV – LAV)	4.319 (2.118–10.368)	0.0003
(V30 – LAV30)∕(TLV – LAV)	5.707 (2.676–14.706)	<0.0001
LAV (cc)	0.758 (0.378–1.351)	0.383
LAV%	0.901 (0.489–1.571)	0.722
TLV – LAV (cc)	0.869 (0.491–1.492)	0.617
MLD (Gy)	3.615 (1.893–7.836)	0.0003
MED (Gy)	1.906 (1.154–3.270)	0.0138
MLWED (Gy)	3.950 (2.042–8.746)	0.0002

The number of symptomatic RP patients / Total patients was 27 / 100

Data were divided by the standard deviation and adjusted for chemotherapy and interstitial lung disease. V2/5/10/20/30 = volume of the lung receiving a dose ≥2/5/10/20/30 Gy, respectively; V2/5/10/20/30% = percentage of lung volume irradiated with ≥2/5/10/20/30 Gy, respectively; LAV2/5/10/20/30 = volume of the lung without low attenuation volume (LAV) receiving 2/5/10/20/30 Gy, respectively; TLV = total lung volume; MLD = mean lung dose; MED = mean emphysema dose; MLWED = mean lung without emphysema dose

In multivariable logistic regression analysis for RP ≥ Grade 3, the results were very similar to those for symptomatic RP, i.e., all of the dosimetric parameters except V2, V5, V10, and MED were significantly related to RP ≥ Grade 3 (Additional file 1).

**Fig. 3:**
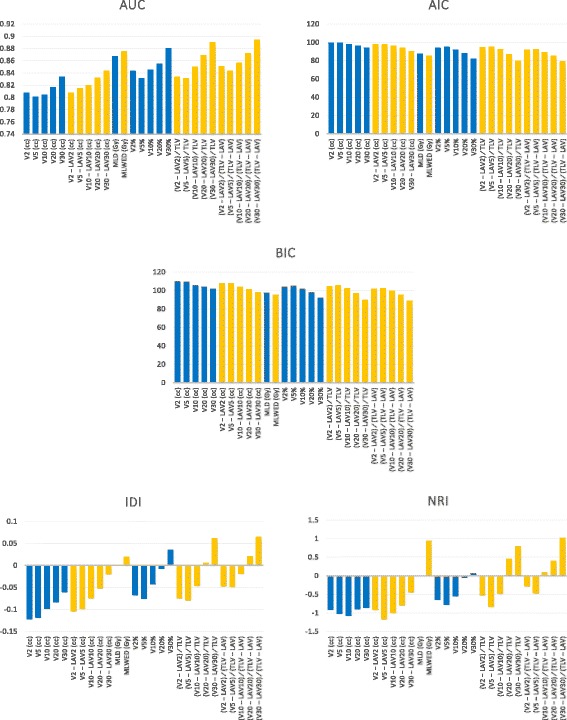
The parameters with smaller AIC value or smaller BIC value are preferable. The parameters with bigger AUC value, IDI value, or NRI value are preferable. Compared with traditional dosimetric parameters (blue bar), counterpart measurements without LAV (orange bar) improved risk prediction of symptomatic RP. Data were adjusted for chemotherapy and interstitial lung disease. AUC = difference in the area under the receiver operating characteristic curve; V2/5/10/20/30 = volume of the lung receiving a dose ≥2/5/10/20/30 Gy, respectively; V2/5/10/20/30% = percentage of lung volume irradiated with ≥2/5/10/20/30 Gy, respectively; LAV2/5/10/20/30 = volume of the lung without low attenuation volume (LAV) receiving 2/5/10/20/30 Gy, respectively; TLV = total lung volume; MLD = mean lung dose; MED = mean emphysema dose; MLWED = mean lung without emphysema dose

The predictive performance of dosimetric parameters for symptomatic RP was compared using AUC, AIC, BIC, IDI, and NRI (Fig. 3). The parameters with smaller AIC value or smaller BIC value are preferable when comparing two or more parameters. The parameters with bigger AUC value, IDI value, or NRI value are preferable when comparing two or more parameters. The same tendency was shown in all statistical measures. The irradiated lung volume (V2, V5, V10, V20, V30) showed a lower predictive performance for symptomatic RP than the counterpart measurements of the lung without LAV (V2 – LAV2, V5 – LAV5, V10 – LAV10, V20 – LAV20, V30 – LAV30). MLWED predicted the risk of symptomatic RP more accurately than MLD. For every lung volume measurement, the ratio of the irradiated lung volume without LAV to the TLV without LAV [(V2 – LAV2) ∕ (TLV – LAV), (V5 – LAV5) ∕ (TLV – LAV), (V10 – LAV10) ∕ (TLV – LAV), (V20 – LAV20) ∕ (TLV – LAV), (V30 – LAV30) ∕ (TLV – LAV)] predicted the risk of symptomatic RP more accurately than the conventional dosimetric parameters, including the V2%, V5%, V10%, V20%, and V30% counterparts. The most accurate dosimetric predictor of symptomatic RP was the ratio of the lung without LAV receiving ≥30Gy to the TLV without LAV (AIC, 78.849; BIC, 88.848; AUC, 0.894; IDI, 0.064; NRI, 1.016). According to the receiver operating characteristic analysis, the threshold value of the (V30 − LAV30) / (TLV − LAV) predictor was 0.161 (sensitivity, 74.1%; specificity, 91.8%).

## Discussion

In this study, we compared various parameters to identify the best predictor for RP in lung cancer patients. First, we found that the absolute lung volume inside the irradiation field was correlated with the occurrence of symptomatic RP (≥ Grade 2). It has been well established that the percentage of irradiated lung volume is correlated with the occurrence of RP [9, 13, 16]. Tsujino et al. proposed that absolute lung volume spared from 5 Gy is significantly associated with RP [7], but absolute lung volume inside the irradiation field has not been evaluated until now. Second, irradiated lung volume without LAV was a better dosimetric predictor of RP than irradiated lung volume including LAV. Third, we identified a new dosimetric parameter that was the most accurate predictor of symptomatic RP: (V30 − LAV30) ∕ (TLV − LAV). These findings suggest that the total amount of the lung volume without emphysematous lesions inside the radiation field might influence the onset of RP.

Various dosimetric parameters including MLD and V20% have been reported to predict RP [9, 11, 13, 16, 17]. The dosimetric parameters analyzed in this study were mutually correlated, therefore we used AUC, AIC, BIC, IDI, and NRI to compare them for RP predictive power. To our knowledge, this is the first report using multiple statistical measures to determine the strongest predictor for RP.

Takeda et al. reported that heavy smoking is the strongest negative predictor of severe RP and is correlated with severe COPD [5]. Wang et al. also noted that lower baseline pulmonary function did not increase the risk of symptomatic radiation-induced lung toxicity [6]. Our results are in line with these reports. Studies of bronchoalveolar lavage in human subjects, and bronchoalveolar lavage and ultrastructural morphology in animal models, also demonstrated that there is less inflammation in the alveolar tissue in those irradiated and exposed to smoking than in those irradiated but not exposed to smoking [18, 19].

By contrast, other authors have argued that COPD and severe pulmonary emphysema are significant risk factors for RP [1, 2]. Inoue et al. investigated the relationship between the diagnosis of COPD and RP [1]. They did not find any association between emphysema volume inside the irradiation volume and RP. However, because the diagnosis of COPD in their report was based on the forced expiratory volume in 1 s (FEV_1_) ∕ forced vital capacity (FVC) < 0.70 ratio, the degree of emphysematous lesions, and especially early changes in the lung, were not evaluated using CT. A prospective study is currently underway to investigate the similar concept of the present study [20].

Chemotherapy regimens including carboplatin/paclitaxel and ILD have also been reported as risk factors for RP [3, 7, 21–23]. In the present study, chemotherapy and ILD were significantly associated with the occurrence of symptomatic RP. Therefore, we thought these factors might be confounding factors and chemotherapy and ILD were used as adjusted factors in the logistic models in this study.

The threshold used for quantification of emphysema is generally −950 HU, which is appropriate for use at full inspiration [24, 25]. In the present study, CT scanning was conducted under free breathing, which is nearly equal to expiratory CT; therefore, we used the −856HU threshold [14, 26–29]. We also validated that the LAV in inspiratory CT was highly correlated with the LAV in CT under free breathing (Fig. 2).

This study had several limitations that warrant further evaluation. First, our subjects were relatively small in number and came from a single institution. This was also a retrospective study. A prospective multicenter study is needed to confirm the results. Second, we did not evaluate ILD quantitatively; however, it should be noted that currently there is no established method for quantitative evaluation of ILD. Third, because many values were missing, we were unable to evaluate pulmonary function tests. Forth, the large number of simultaneous independent variables were used for multivariate logistic regression in comparison to the sample size. Therefore they can be prone to overfitting.

## Conclusions

We conclude that use of irradiated lung volume without emphysema leads to more accurate dosimetric prediction of RP than traditional parameters. The most accurate dosimetric predictor of RP was the ratio of the lung without LAV receiving ≥30 Gy to the TLV without LAV.

## Additional file

Additional file 1: Table S1. Multivariate logistic regression analysis for radiation pneumonitis ≥ Grade 3. (DOCX 19 kb)

## Abbreviations

AIC: Akaike’s information criterion
AUC: Area under the receiver operating characteristic curve
BIC: Bayesian information criterion
BMI: Body mass index
COPD: Chronic obstructive pulmonary disease
CT: Computed tomography
G2: Grade 2
G3: Grade 3
IDI: Integrated discrimination improvement
ILD: Interstitial lung disease
IMRT: Intensity-modulated radiotherapy
LAV: Low attenuation volume
LAV%: The percentage of low attenuation volume to total lung volume
MED: Mean emphysema dose
MLD: Mean lung dose
MLWED: Mean lung without emphysema dose
NRI: Net reclassification improvement
RP: Radiation pneumonitis
RT: Radiotherapy
TLV: Total lung volume
V2: The volume of the lung receiving a dose of ≥2 Gy
V5: The volume of the lung receiving a dose of ≥5 Gy
V10: The volume of the lung receiving a dose of ≥10 Gy
V20: The volume of the lung receiving a dose of ≥20 Gy
V30: The volume of the lung receiving a dose of ≥30 Gy
V2 – LAV2: The volume of the lung without low attenuation volume receiving a dose of ≥2 Gy
V5 – LAV5: The volume of the lung without low attenuation volume receiving a dose of ≥5 Gy
V10 – LAV10: The volume of the lung without low attenuation volume receiving a dose of ≥10 Gy
V20 – LAV20: The volume of the lung without low attenuation volume receiving a dose of ≥20 Gy
V30 – LAV30: The volume of the lung without low attenuation volume receiving a dose of ≥30 Gy
V2%: The percentage of lung volume irradiated with ≥2 Gy
V5%: The percentage of lung volume irradiated with ≥5 Gy
V10%: The percentage of lung volume irradiated with ≥10 Gy
V20%: The percentage of lung volume irradiated with ≥20 Gy
V30%: The percentage of lung volume irradiated with ≥30 Gy
(V2 – LAV2) / (TLV – LAV): The ratio of the lung without low attenuation volume receiving ≥2 Gy to the total lung volume without low attenuation volume
(V5 – LAV5) / (TLV – LAV): The ratio of the lung without low attenuation volume receiving ≥5 Gy to the total lung volume without low attenuation volume
(V10 – LAV10) / (TLV – LAV): The ratio of the lung without low attenuation volume receiving ≥10 Gy to the total lung volume without low attenuation volume
(V20 – LAV20) / (TLV – LAV): The ratio of the lung without low attenuation volume receiving ≥20 Gy to the total lung volume without low attenuation volume
(V30 – LAV30) / (TLV – LAV): The ratio of the lung without low attenuation volume receiving ≥30 Gy to the total lung volume without low attenuation volume
