# Supplementary material for: Exclusion of emphysematous lung from dose-volume estimates of risk improves prediction of radiation pneumonitis
Source: Radiat Oncol. 2017 Oct 2;12:160. doi: 10.1186/s13014-017-0891-z (PMC5625816; doi:10.1186/s13014-017-0891-z)
Supplement: Additional file 1: Table S1. — Multivariate logistic regression analysis for radiation pneumonitis ≥ Grade 3. (DOCX 19 kb) [file 13014_2017_891_MOESM1_ESM.docx]

Additional file 1

Table S1 Multivariate logistic regression analysis for radiation pneumonitis ≥ Grade 3.

| Parameter | Odds ratio | P Value | |
| --- | --- | --- | --- |
| V2 (cc) | 1.701 (0.936­­­­­­­­­­­–3.104) | 0.0763 | |
| V5 (cc) | 1.350 (0.746­­­­­­­­­­­–2.420) | 0.3099 | |
| V10 (cc) | 1.471 (0.814­­­­­­­­­­­–2.641) | 0.1910 | |
| V20 (cc) | 1.701 (0.936­­­­­­­­­­–3.104) | 0.0763 | |
| V30 (cc) | 2.185 (1.179­­­­­­­­­­­–4.251) | 0.0145 | |
| V2 − LAV2 (cc) | 1.708 (0.939­­­­­­­­­­­–3.270) | 0.0866 | |
| V5 − LAV5 (cc) | 1.649 (0.910­­­­­­­­­­­–3.140) | 0.1052 | |
| V10 − LAV10 (cc) | 1.799 (0.993­­­­­­­­­­­–3.459) | 0.0592 | |
| V20 − LAV20 (cc) | 2.094 (1.131­­­­­­­­­­­–4.180) | 0.0237 | |
| V30 − LAV30 (cc) | 2.767 (1.409­­­­­­­­­­­–6.081) | 0.0057 | |
| LAV2 (cc) | 0.744 (0.294–1.416) | | 0.449 |
| LAV5 (cc) | 0.652 (0.234–1.311) | | 0.325 |
| LAV10 (cc) | 0.615 (0.208–1.291) | | 0.296 |
| LAV20 (cc) | 0.684 (0.232–1.385) | | 0.405 |
| LAV30 (cc) | 0.909 (0.365–1.616) | | 0.795 |
| V2% | 1.924 (1.027­­­­­­­­­­­–3.828) | 0.0480 | |
| V5% | 1.701 (0.921­­­­­­­­­­­–3.276) | 0.0954 | |
| V10% | 1.869 (1.009­­­­­­­­­­­–3.645) | 0.0522 | |
| V20% | 2.316 (1.212­­­­­­­­­­–4.764) | 0.0146 | |
| V30% | 3.567 (1.701­­­­­­­­­­­–8.658) | 0.0019 | |
| (V2 − LAV2) ∕ TLV | 2.244 (1.178­­­­­­­­­­­–4.664) | 0.0191 | |
| (V5 − LAV5) ∕ TLV | 1.999 (1.077­­­­­­­­­­–3.952) | 0.0333 | |
| (V10 – LAV10) ∕ TLV | 2.216 (1.186­­­­­­­­­­­–4.503) | 0.0172 | |
| (V20 – LAV20) ∕ TLV | 2.778 (1.426­­­­­­­­­­­–6.083) | 0.0050 | |
| (V30 – LAV30) ∕ TLV | 4.214 (1.944­­­­­­­­­­­–10.907) | 0.0009 | |
| (V2 − LAV2) ∕ (TLV − LAV) | 2.264 (1.190­­­­­­­­­­­–4.669) | 0.0176 | |
| (V5 – LAV5) ∕ (TLV − LAV) | 2.062 (1.102­­­­­­­­­­­–4.120) | 0.0289 | |
| (V10 – LAV10) ∕ (TLV − LAV) | 2.268 (1.202­­­­­­­­­­­–4.638) | 0.0157 | |
| (V20 − LAV20) ∕ (TLV − LAV) | 2.831 (1.444­­­­­­­­­­­–6.133) | 0.0042 | |
| (V30 – LAV30) ∕ (TLV − LAV) | 4.273 (1.983­­­­­­­­­­4–10.852) | 0.0007 | |
| LAV (cc) | 0.566 (0.179­­­­­­­­­­­–1.257) | 0.234 | |
| LAV% | 0.678 (0.271­­­­­­­­­­­–1.400) | 0.339 | |
| TLV – LAV (cc) | 0.813 (0.414­­­­­­­­­­­–1.572) | 0.538 | |
| MLD (Gy) | 2.491 (1.303­­­­­­­­­­–5.111) | 0.0080 | |
| MED (Gy) | 1.554 (0.872­­­­­­­­­­­–2.750) | 0.1259 | |
| MLWED (Gy) | 2.793 (1.444­­­­­­­­­­­–5.879) | 0.0036 | |

The number of RP ≥ Grade 3 patients / Total patients was 15 / 100.

Data were divided by the standard deviation and adjusted for chemotherapy and interstitial lung disease. V2/5/10/20/30 = volume of the lung receiving a dose ≥ 2/5/10/20/30 Gy, respectively; V2/5/10/20/30% = percentage of lung volume irradiated with ≥ 2/5/10/20/30 Gy, respectively; LAV2/5/10/20/30 = volume of the lung without low attenuation volume (LAV) receiving 2/5/10/20/30 Gy, respectively; TLV = total lung volume; MLD = mean lung dose; MED = mean emphysema dose; MLWED = mean lung without emphysema dose.
